# Evaluating a novel team learning approach for integrating drugs in sports education in preclinical medical training

**DOI:** 10.3389/fmed.2025.1480967

**Published:** 2025-02-10

**Authors:** Samuel Pelobello, Grayson Potter, Daniel K. Rogstad, Andrew J. Mock, Sean M. Wilson

**Affiliations:** ^1^Lawrence D. Longo, MD Center for Perinatal Biology, Department of Basic Sciences, Loma Linda University School of Medicine, Loma Linda, CA, United States; ^2^Elite Education, United States Anti-Doping Agency, Colorado Springs, CO, United States; ^3^Department of Medical Education, Loma Linda University School of Medicine, Loma Linda, CA, United States; ^4^Preventative Medicine, Loma Linda University School of Medicine, Loma Linda, CA, United States

**Keywords:** performance-enhancing drugs, supplements, case-base team learning, medical education, drugs in sports

## Abstract

Use of performance-enhancing drugs and supplements continues to be pervasive in sports. Medical practitioners are key because they are well positioned to prevent doping among athletes as they are a trusted resource for the patients whom they serve. At Loma Linda University School of Medicine, we are seeking to provide medical students with education on the topic of drugs in sports so that they can better serve their patients. This study evaluated the implementation of a novel Case-Based Team Learning session on drugs in sports for preclinical first year (MS1) and second year (MS2) medical students. The session aimed to introduce fundamental concepts of performance-enhancing drugs, anti-doping regulations, and patient communication strategies within the context of sports medicine. Post instructional survey data on the learning environment and qualitative feedback responses were collected from 189 MS1 and 170 MS2 students. Results of the quantitative data showed that MS1 students rated the session more positively than MS2 students. Qualitative data was collected through open-ended questions, allowing for more detailed and nuanced responses. AI models were used to identify common themes and patterns in the qualitative feedback responses. These responses provided valuable insights for future curriculum refinement and development of the newly implemented drugs in sports education program. Both cohorts appreciated the interactive nature of the session and real-life applications but identified areas for improvement, including better alignment with curriculum objectives and exam preparation. Key challenges included balancing content relevance with broader medical education goals and integrating communication skills training within a large group setting. Faculty reflection highlighted the need for restructuring the session to better match instructional block content and USMLE Step 1 exam preparation. Future iterations will focus on emphasizing drug pharmacology, mechanisms of action, and physiological effects for MS1 students, while providing opportunities for more comprehensive knowledge integration through the case studies for MS2 students. This evaluation of the learning session underscores the importance of iterative curriculum development in medical education, particularly when introducing novel topics like drugs in sports.

## 1 Introduction

Doping is a banned practice in many competitive sports that should be part of both preclinical and clinical medical education. In the medical curricula, it serves as an effective tool to teach drug mechanism of action, patient interaction, harm prevention, and medical ethics. Early education on doping prepares future medical practitioners to protect athletes from prohibited substances and safeguards the medical profession from issues stemming from inadequate knowledge of the topic.

Understanding doping in sports and medicine requires knowledge of its historical and societal context, as well as its evolution. In 1963, doping was first officially defined as the use of foreign substances to enhance performance (1). The word “doping” likely originates from the term “dop”, used by various African tribes, which referred to a drink that enhanced physical attributes. The practice of doping, however, can be traced back to as early as the ancient Mesopotamian and Egyptian civilizations, which used opiates to improve physical performance (2). Modern doping emerged in the second half of the twentieth century and led to tragic incidents such as that of Knud Enemark Jensen. He was a Danish cyclist whose amphetamine use contributed to his fatal collapse during the 1960 Rome Olympic Games (3). Jensen's death, along with other incidents, led the International Olympic Committee to form a medical committee in 1961 and contributed to the institution of drug testing at the 1968 Winter and Summer Olympics (4).

Despite anti-doping efforts, doping intensified in the 1980s and 1990s. This frustrated sports officials, fans, and sponsors. The 1998 Tour de France scandal, involving systemic drug use by the Festina Professional Cycling Team, led to the formation of the World Anti-Doping Agency (WADA) in 1999 (5). Since the formation of WADA, athlete testing, sanctions, and education have increased. This is thought to have helped curtail the use of performance-enhancing drugs, though doping continues to be a significant issue. A study on Italian athletes showed that between 2.8% and 4.8% engaged in doping from 2003 to 2013 (6). More recent data from the United States Anti-Doping Agency (USADA) indicates that in 2022, 0.77% of the 256,769 samples tested had adverse analytical findings. These findings suggest that these athletes had used banned substances or methods (7). Notably the absence of a positive drug test does not necessarily mean an athlete has not doped; but rather they may simply not have been caught. Consequently, many doping cases likely go undetected (8).

One critical question is why does doping persist in sports despite advances in knowledge, testing technology, and education about performance-enhancing drugs? Overcoming doping in sports faces major hurdles, stemming from the diverse reasons athletes use performance-enhancing substances. While many assume athletes dope primarily to boost performance, the reality is more complex and multifaceted. In some instances, coaches and managers coerce athletes to use banned substances to increase performance (9). More commonly, however, athletes inadvertently take prohibited substances due to use of dietary supplements (10). On occasion, athletes may also take prohibited substances by using prescribed or over the counter medications (10, 11). Medical practitioners play a crucial role in preventing many forms of doping, as athletes consider them knowledgeable and trusted resources.

Physicians and other healthcare providers play a crucial role in an athlete's career, overseeing their general health and well-being while managing both acute and chronic injuries or diseases. However, medical practitioners working with athletes often face challenges in understanding the full implications of prescribing medications to their athletic patients (12). The WADA code and its annual prohibited list define the substances that are banned in sports (13). The frequent updates to the WADA code and prohibited list further complicate the task of tracking prohibited substances for prescribing physicians. This difficulty is compounded by the fact that consideration of prohibited substances is not typically a primary concern for practitioners when prescribing medicines to improve the health and well-being of their patients. As a result, there is often a lack of understanding among healthcare providers regarding the potential impact of their prescriptions on an athlete's eligibility to compete.

Medical practitioners can also face ethical dilemmas when treating athletes, for which they may be ill equipped. This includes providing support to reduce harm in athletes who knowingly use banned substances and providing appropriate care for medical conditions while avoiding positive doping tests. Such scenarios require balancing ethical duties, athlete welfare, and anti-doping compliance. The lack of understanding is illustrated in survey-based studies. One study performed on French General practitioners (GPs) found that most thought doping was a problem, but 83% indicated that they did not believe they had sufficient training regarding performance-enhancing drugs (14). A similar study conducted in 771 GPs in Ireland discovered that 92% of the practitioners felt they had a role in preventing doping although only 9% thought they had adequate training (15).

Medical practitioners need to understand performance-enhancing substance use and misuse due to the severe consequences athletes face for positive doping tests. Under WADA guidelines, a first offense can result in a 2-year competition ban, while a second offense may lead to a lifetime suspension (13). Athletes have faced penalties for prescribed medications despite no intent to enhance performance. In one case, a wheelchair athlete received a 2-year ban after testing positive for a prescribed stimulant, even though a panel agreed she had no performance-enhancing intent (16). The case of 16-year-old gymnast Andreea Raducan further illustrates the complexities of doping regulations. Raducan was stripped of her Olympic gold medal after testing positive for pseudoephedrine, an ingredient in an over-the-counter cold medication provided by the team doctor (17). Despite the adjudication panel agreeing that she needed the medication and no party was at fault, the medal was forfeited as the substance was prohibited. These incidents underscore the need for proper physician education on doping, covering prescription, over-the-counter medications, and supplements. Lack of knowledge can not only jeopardize an athlete's career but also negatively impact their overall health and wellness. Relatedly, physicians need to understand the Therapeutic Use Exemption process as it ensures athletes who medically require prohibited medications receive appropriate support and treatment (18).

**Figure 1:**
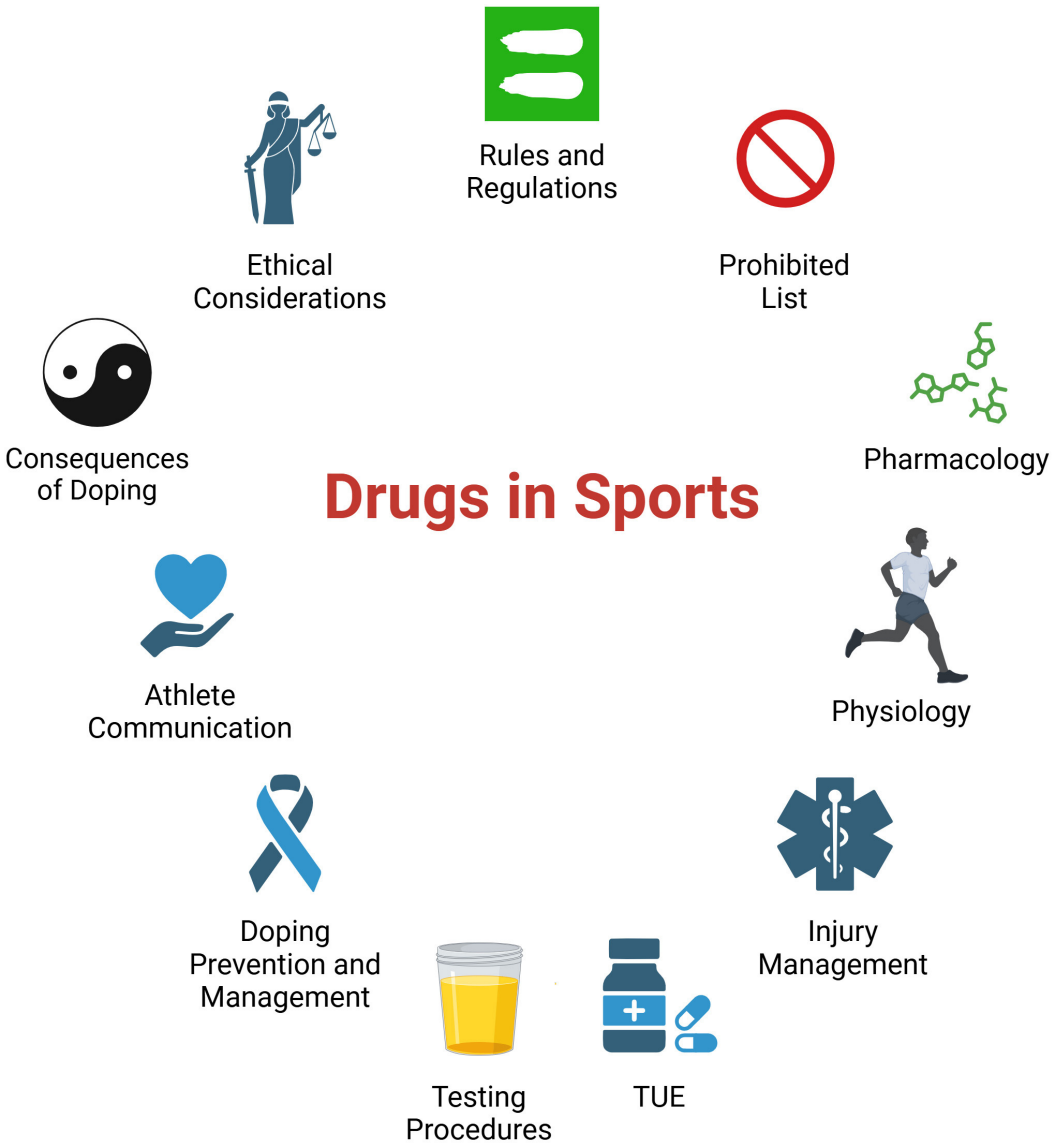
Illustration of the interconnected aspects of anti-doping education, highlighting the complex knowledge base required for medical professionals in sports medicine. Specialized courses in antidoping education can encompass multiple areas for healthcare professionals working with athletes. The educational framework includes understanding anti-doping rules and regulations, the substances and methods prohibited in sport, pharmacology, and physiology. The courses cover aspects related to injury management strategies and training on the Therapeutic Use Exemption (TUE) process. These courses also cover testing procedures, doping prevention and management techniques and effective athlete communication. There is education on the consequences of doping, both for the athlete's health and career. Curriculum also addresses the ethical considerations surrounding doping in relation to patient care, emphasizing the importance of balancing medical needs with sports integrity (19–21, 29). Created in BioRender. Wilson, S. (2025) https://BioRender.com/q95s032.

Class sessions regarding drugs in sports are rarely included in preclinical or clinical curricula (19). Instead the topic is typically taught in specialized courses designed for medical practitioners who work directly with athletes (20, 21). To address this gap, we have introduced doping education during the preclinical years. However, the scarcity of curricula for professional students makes it unclear which aspects of doping education (see Figure 1) should be prioritized and to what depth. The purpose of this manuscript is to evaluate our inaugural teaching session on drugs in sports, highlighting the goals of our session, providing student and faculty feedback, and a plan of action so that we can improve student learning.

## 2 Pedagogical framework, learning environment, and methodology

### 2.1 Pedagogical framework

We have implemented a comprehensive approach to educate medical students at Loma Linda University School of Medicine that aims to enhance the ability of our students to serve patients effectively. The pedagogical framework for large classroom sessions incorporates a diverse range of learning methodologies. These strategies cater to different learning styles and optimize knowledge retention. This includes traditional didactic lectures, team-based learning, flipped classroom models, practical demonstrations, and case-based learning. For the drugs in sports curriculum, we specifically adopted a “case-based team learning” (CBTL) approach. In the CBTL format, one professor facilitates a large group session where students work in teams of 4 to 6 to analyze and solve real-world case studies.

We chose to use a case-based format in teams to teach drugs in sports for a number of reasons. This learning strategy maximizes student engagement with the material and fosters collaborative problem-solving skills (22). This strategy also enhances critical thinking and promotes peer-to-peer learning above didactic learning methods. Case-based learning also provides a practical context for understanding the complexities of working with athlete populations and understanding drugs in sports. The underlying premise is that by engaging with realistic scenarios, students develop the skills necessary to navigate the ethical and medical challenges they may encounter in their future practice. Ultimately this prepares students to better serve their patients in this critical area of sports medicine.

### 2.2 Learning environment

The case-based team learning environment for both MS1 and MS2 students was tailored to their respective stages in the medical curriculum. For MS1 students, the session was integrated into an organ-systems block focusing on endocrinology and reproduction. The session was strategically placed after lectures on catecholamines and steroid hormones to reinforce and build on existing knowledge. MS2 students, on the other hand, encountered this material during a summary topics and multisystem integration block at the conclusion of their second year. This allowed for a more comprehensive application of their accumulated knowledge. Both cohorts were presented with case studies designed to challenge their understanding of doping.

MS1 students analyzed three cases: a strongman athlete knowingly using anabolic steroids, an Olympic female swimmer with polycystic ovary syndrome (PCOS) receiving prescribed medications, and a teenage wrestler using over-the-counter supplements with potential banned substances or side effects (23) (see Table 1). MS2 students were given an additional case study focusing on a female marathon runner presenting with relative energy deficiency in sport (RED-S) (24–26). The RED-S case was omitted from the MS1 session due to time constraints, balancing comprehensive coverage with practical limitations in curriculum design.

**Table 1:** List of cases used in the CBTL.

Case 1	An elite-level male athlete is preparing for a strongman competition. He comes to you asking about issues related to repeated injections of an anabolic steroid he has been using. He follows this by saying “When you want to be the best, you do whatever it takes.”
Case 2	An Olympic-level female swimmer is being treated for symptoms involving polycystic ovary syndrome (PCOS). She has questions regarding her treatment and has come to you as you are the head doctor for USA Swimming. Her regular OB/GYN prescribed clomiphene, semaglutide, and metformin to help with the symptoms. You are discussing her options and trying to assess the best course of action.
Case 3	A 17-year-old male wrestler from Redlands High School comes to your clinic complaining of an inability to focus or sleep. As part of your discussion, you discover that they are taking several different supplements including Jack 3D from USP Labs, Godzilla from Lawless Labs, and Creatine. They also drink Monster energy drinks each morning and before practice in the afternoon.
Case 4 (MS2s only)	A world-class level female marathon runner presents with amenorrhea. She has frequent mild upper respiratory infections. She is depressed and has trouble falling asleep. Her LH, FSH, and estradiol levels are all below normal.

### 2.3 Data collection methods

This quality improvement project did not meet the criteria for human subject research as determined by the Institutional Review Board at Loma Linda University. The study focused on evaluating performance-enhancing drugs education in the 2023–2024 preclinical curriculum. The study targeted 189 first-year (MS1) and 170 second-year (MS2) medical students. Data collection methods were designed to gather comprehensive feedback on the newly implemented learning sessions and the engagement of the student learners.

### 2.4 Tools used

An anonymous standardized survey served as the primary data collection tool, administered to students after completing the instructional block. Students could complete the survey remotely but had to submit responses before grade release. This ensured high participation while preserving anonymity. The survey was identical for both MS1 and MS2 cohorts and is used universally to assess all preclinical learning sessions and instructors (Table 2). It evaluates instructional content, delivery mode, and overall teaching quality. The survey aims to gather comprehensive insights into students' perceptions of the new curriculum, providing valuable feedback for future improvements to the educational program.

**Table 2:** MS1 and MS2 student evaluations.

**Teaching skills**										
		**None**	**< 25%**	**25–50%**	**51–75%**	**>75%**		**Mean**	**SD**	
I attended at least the following percentage of lectures by this faculty member:	MS1	11.11%	9.52%	4.76%	11.11%	63.49%		4.06	1.4	ND
	MS2	14.71%	3.53%	4.71%	6.47%	70.59%		4.15	1.5	
		**Strongly disagree**	**Disagree**	**Uncertain**	**Agree**	**Strongly agree**	**N/A**			
The teacher made efficient use of the allocated time.	MS1		0.53%	7.94%	49.74%	38.10%	3.70%	4.3	0.6	*P < * 0.0001
	MS2		5.88%	14.12%	44.12%	25.29%	10.59%	3.99	0.8	
The audiovisual aids (e.g., powerpoint, animations, heart sounds, film clips, etc.) augmented the teacher's presentation.	MS1	1.06%	1.59%	6.88%	47.62%	39.15%	3.70%	4.27	0.8	*P < * 0.001
	MS2		4.12%	11.76%	49.41%	24.12%	10.59%	4.05	0.8	
The teacher's presentation(s) added value to the syllabus/handout.	MS1		2.65%	6.35%	48.15%	39.15%	3.70%	4.29	0.7	*P < * 0.0001
	MS2		4.12%	14.71%	44.12%	24.12%	12.94%	4.01	0.8	
The organization of the presentation made it easy to follow.	MS1	0.53%	2.12%	7.94%	48.15%	38.62%	2.65%	4.26	0.7	*P < * 0.0001
	MS2	1.76%	2.35%	14.71%	48.24%	22.35%	10.59%	3.97	0.8	
The teacher's handout captured the most salient points of the presentation.	MS1	1.59%	3.17%	7.94%	43.39%	39.15%	4.76%	4.21	0.9	*P < * 0.0001
	MS2	1.18%	7.65%	14.12%	40.59%	23.53%	12.94%	3.59	0.9	
The teacher provided learning objectives.	MS1		0.53%	5.29%	47.09%	43.92%	3.17%	4.39	0.6	*P < * 0.0001
	MS2	0.59%	3.53%	12.35%	48.24%	24.71%	10.59%	4.04	0.8	
The teacher explained the clinical relevance of the material being discussed.	MS1			7.94%	43.92%	44.97%	3.17%	4.38	0.6	*P < * 0.0001
	MS2		1.18%	9.41%	51.76%	27.06%	10.59%	4.17	0.7	
**Interpersonal relationships**										
		**Strongly disagree**	**Disagree**	**Uncertain**	**Agree**	**Strongly agree**	**N/A**	**Mean**	**SD**	
The teacher showed care and concern for my learning.	MS1		0.53%	6.35%	38.62%	47.62%	6.88%	4.43	0.6	*P < * 0.001
	MS2		0.59%	7.65%	48.24%	29.41%	14.12%	4.24	0.6	
The teacher consistently challenged me to take responsibility for my own learning.	MS1		0.53%	5.82%	41.27%	44.44%	7.94%	4.43	0.6	*P < * 0.001
	MS2		0.59%	8.82%	48.82%	27.06%	14.71%	4.2	0.6	
**Overall effectiveness**										
		**Unsatisfactory**	**Below**	**At expectation**	**Above**	**Outstanding**	**N/A**			
I would rate the overall effectiveness of this instructor's presentation(s) as:	MS1		3.70%	32.80%	23.28%	37.04%	3.17%	3.97	0.9	*P < * 0.001
	MS2	0.59%	6.47%	33.53%	27.06%	21.76%	10.59%	3.7	0.9	

MS1 *N* = 189; MS2 *N* = 170. Surveys utilized a 5-point Likert scale, complemented by a “not applicable” option. Statistical analysis comparing first-year (MS1) and second-year (MS2) medical student survey responses employed the Mann-Whitney U test, with significance set at *P* < 0.05.

### 2.5 Analysis methods

Student feedback on the learning environment was analyzed using mixed-methods, combining quantitative and qualitative data collection methods. Quantitative data was gathered through standardized questions using a Likert scale, with results summarized and presented in Table 2. Comparisons in composite scores between MS1 and MS2 students were made using a Mann Whitney U test, with a critical cutoff of *P* < 0.05 for statistical significance using GraphPad Prism 10.4.0 (San Diego, CA). Qualitative data was collected through open-ended questions, allowing for more detailed and nuanced responses. These responses are provided in Appendix 1. There was a total of 31 open-ended responses from MS1 students and 16 from MS2 students. To identify common themes and patterns in qualitative feedback, several AI models were utilized through Perplexity Pro, including the Perplexity Pro search, GPT-4o by OpenAI, and Claude 3.5 Sonnet by Anthropic. This comprehensive approach provided valuable insights for future curriculum refinement and development of the newly implemented drugs in sports education program, offering a thorough evaluation of student experiences and perceptions.

## 3 Learning objectives

The session objectives shown in Table 3 included several but not all topics in performance-enhancing drugs (PEDs) and sports medicine (Figure 1). These topics are presented and prioritized by importance and aligned with case studies. These included defining the WADA Prohibited List, understanding a physician's role in PED situations, explaining therapeutic use exemptions, and identifying risks of dietary supplements. MS2 students had additional objectives on RED-S (24, 27, 28). The longer MS2 session also explored PEDs in greater depth, covering widely used substances, the WADA list update process, positive tests in each substance group with real-world examples, and general sports drug use statistics.

**Table 3:** CBTL learning objectives.

LO1	Define the criteria for a substance or method to be included on the World Anti-doping Agency's Prohibited List. Identify the substances and methods that are on the Prohibited List. Know the prevalence of use. Utilize the USADA resources to check the status of medications (36). Understand the differences between in-competition and out-of-competition periods and substances and methods not permitted during those periods.
LO2	Describe the role of the practitioner in Performance-enhancing Drugs. Identify key aspects of the anti-doping code. Know what resources are available. Identify best practices for the clinician.
LO3	Explain the purpose of a Therapeutic Use Exemption (TUE). Know the various tools available through the U.S. Anti-Doping Agency (USADA) to assist the athlete-patient in the process. Understand the medical providers' responsibilities in helping the athlete-patient submit a complete TUE.
LO4	Identify both medical and anti-doping risks that accompany dietary supplement products. Identify key substances found in supplements. Identify “red flags” in supplements (23). Know the tools that are available through USADA regarding supplements including the High-Risk List and third-party certification information.

The sessions primarily focused on developing effective patient communication skills regarding substance use and misuse. This approach aimed at equipping students with an understanding of the multifaceted landscape of PEDs in sports medicine, balancing theoretical knowledge with practical applications and communication skills. We deliberately minimized detailed discussions on drug pharmacology and their mechanisms of action to focus on practical applications. We also limited discussion on doping control and testing procedures, rules and regulations, doping prevention and management strategies, and other aspects found in specialized courses designed for medical practitioners (19–21, 29). Students were encouraged to apply previously learned communication techniques, such as motivational interviewing, when addressing the case study questions (see Table 4).

**Table 4:** List of group questions for each case.

	**Red**	**Blue**	**Green**
Case 1	What are your responsibilities and obligations?	What tests and physical exams would you do?	What is the course of action?
Case 2	Which of these drugs are permitted in- competition/out-of-competition?	Are there any alternative treatment strategies?	What documentation is needed for you to provide a therapeutic use exemption (TUE)?
Case 3	How do you discuss the use of supplements?	What are the risks and benefits associated with using these supplements?	Which of the compounds in the supplements are permitted in-competition/out-of- competition?
Case 4	What is the likely underlying cause?	What are the pharmacological treatments?	What are the alternative treatments?

The sessions were designed to foster collaborative learning and active engagement with the course material. The class was organized into small groups of 4–6 students, resulting in 21 MS1 and 20 MS2 groups. These groups were assigned colors (Red, Blue, or Green) based on their classroom location. Each case had three questions, with groups tasked to answer one question per case according to their color designation, as outlined in Table 4. Students were given time to research their questions using various resources, including lecture slides, PubMed, Google, and AI tools such as ChatGPT. After performing research, the groups convened and discussed the topics. Teams electronically submitted a written report of their analyses after the session, which was evaluated on a participation basis. This structured approach of focused small-group collaboration and broader class discussions created a dynamic and interactive learning environment.

## 4 Results

### 4.1 Student feedback

Based on survey responses from MS1 and MS2 medical students regarding the drugs in sports CBTL session, several common themes emerged. This included both positive elements and areas to improve. The survey responses and themes are detailed in the Appendix materials, which were summarized through AI based approaches. Overall, students found the lecture and CBTL session enjoyable, interesting, and interactive. They appreciated connecting concepts to real-life situations. This is exemplified by the comment, “I actually enjoyed this! It was nice to have something tie into stuff out of the classroom!” Students valued Dr. Wilson's personal experiences and stories, which helped illustrate concepts, and his evident enthusiasm and concern for student learning.

Students identified key areas for improvement. Many expressed difficulties identifying central points and testable information, requesting more explicit learning objectives and emphasis on important takeaways. Students asked for better study aids including comprehensive lecture notes, handouts, or detailed PowerPoint slides. This is summed up well by a student who commented “Please have a handout or more thorough powerpoint, it is difficult to study for quizzes and exams without more detailed notes”. Some students found the presentation and CBTL disorganized or chaotic. They suggested that the structure of the session be improved.

Relevance was a concern, particularly among MS2 students, who questioned the content's applicability to their medical education and USMLE Step 1 exam preparation. A student commented, “While I appreciated this lecture very much and found it to be interesting, I don't think that most of it was relevant to Step 1 and I feel like more of an emphasis of topics that were relevant to our board exam would have been more helpful.” Some felt the material was more suited for athletes or coaches than medical students, with one student commenting “This lecture seems more targeted to athletes, coaches, and trainers, and felt like it had very little relevance to us.”

Time management was a concern. Students suggested that materials could be covered more efficiently, allowing for greater focus on board-relevant topics. This is summarized well by the comment, “The information we saw in class was interesting and relevant, but there were too many materials and activities given to us in a short amount of time. Either lowering the amount of materials or activities would make the class easier to follow.” One individual raised a concern about potentially insensitive comments regarding ADHD and transgender athletes, indicating a need for improved cultural sensitivity.

Quantitative evaluation scores for MS1 and MS2 students are shown in Table 2. Overall, MS1 students rated the teaching skills, interpersonal relationships, and overall effectiveness of the session more positively than MS2 students who were more ambivalent about the session (*P* < 0.001, two-tailed Mann-Whitney U test). Notably, the MS2 students gave lower scores for all relevant questions.

### 4.2 Faculty reflection

From a faculty perspective, the drugs in sports CBTL session had both strengths and areas for improvement. The learning objectives effectively covered essential doping topics through mandatory sessions and interactive group activities, ensuring all students were exposed to crucial material. Inclusion of both performance-enhancing drugs and supplements was considered important because of their widespread use in athletic and non-athletic populations (10). Case study questions effectively introduced the potential for negative side effects of doping substances. Additionally, the lecture introduced students to the Stanford Continuing Medical Education *HealthPro Advantage: Anti-Doping Education for the Health Professional (CME)* (20), which is a valuable resource for further learning about anti-doping practices, communication strategies, therapeutic use exemptions, and the clinician's role (30).

Faculty identified several areas for improvement. MS1 students struggled with patient engagement strategies, likely due to their limited experience with motivational interviewing techniques compared to MS2 students. Furthermore, there was a noticeable mismatch between the session's learning objectives (Table 3) and the overall block objectives for both MS1 and MS2 cohorts. The MS1 session, taught during the endocrinology and reproduction block, and the MS2 session, occurring during the integration block and intensive USMLE Step 1 exam preparation, could have been better aligned with their respective block objectives to enhance relevance and integration within the curriculum.

## 5 Discussion

The inaugural drugs in sports CBTL session for medical students received mixed feedback, highlighting both strengths and areas for improvement in this novel addition to the preclinical curriculum. The session effectively covered essential doping topics through interactive activities, addressing performance-enhancing drugs and supplements. Case studies successfully highlighted common scenarios that practitioners would encounter, and students were introduced to valuable anti-doping resources.

As with many first-time implementations, the session could be improved. Student feedback along with faculty reflection provides valuable insights for refining this new topic's integration into the medical curriculum, emphasizing the need for improved alignment, enhanced relevance, and better session structure. The session needs to be revised to align the content with overall block objectives. Associated with this is to ensure that the session is adjusted to be relevant to their stage of learning and USMLE Step 1 preparation with more efficient coverage of board-relevant topics.

The drugs in sports CBTL session was developed as an introduction to a unconventional topic in medical learning. We incorporated active learning strategies that encompassed working in small groups and class wide discussion that were specifically designed to ensure students engaged with the novel content. To maximize student learning we highlighted specific elements related to doping in sport that would be of use for medical practitioners. This includes those who work with a youth, high school, and recreational athletes up through professionals and those participating in the international arena. Although our approach is innovative, it aligns with the growing trend in medical and pharmacy education to provide comprehensive anti-doping training. This is illustrated by the CME based program offered through Stanford University School of Medicine (20) and the sports pharmacy program offered at the USC Mann School of Pharmacy (19).

The single 1-h session we developed overlaps with classes that introduce students to drugs of abuse, reflecting the broader scope of substance-related issues in healthcare and interrelationship with doping. Our session incorporated motivational interviewing strategies, a key skill for medical practitioners working with patients who suffer from a wide range of substance use disorders (31). This holistic approach mirrors the comprehensive nature of anti-doping education programs developed in partnership with WADA that focuses on preparing medical practitioners to work with Olympic level athletes and teams and covers the technical aspects of anti-doping regulations (29), or the even more intensive international postgraduate program for healthcare professionals offered through the International Olympic Committee (IOC) Medical and Scientific Commission (21). The NCAA Sport Science Institute (32) is another type of resource for medical practitioners as it focuses on athlete education and the broader context of substance use in sports.

The CBTL session integrated multiple elements to help provide future medical professionals context so that they can effectively support athletes at all levels while addressing the complex issues surrounding drugs in sports. This approach was used to equip future healthcare providers with the knowledge and skills necessary to maintain sporting integrity. Further, the session promoted athlete health and well-being, aligning with the current educational trends in sports medicine and anti-doping practices.

Our approach to teaching about drugs in sports in our preclinical program differs significantly from specialized continuing medical education courses, such as the online HealthPro advantage CEU offering from Stanford (20), the WADA-affiliated ADEL course (29), or the more intensive IOC certificate program (21). Our primary objective is to provide foundational knowledge and increase awareness among medical students. We are less concerned with creating authoritative experts on doping. Introducing drugs in sports and its relevance to future medical practitioners, we provide a foundation and spark interest for students who may choose to pursue further specialization in this area. This aligns with our educational philosophy of offering survey-style learning sessions throughout the pre-clinical and clinical years.

We do offer various elective courses to our medical students that enhance their knowledge and practical skills in various topics and specialty areas. However, our perspective is that a comprehensive course on drugs in sports covering all of the elements of Figure 1 would be more appropriate for a sports medicine specialization. This approach ensures that medical students gain a basic understanding of doping, preparing them for potential encounters with sports-related drug use in their future practice, while reserving in-depth, comprehensive coverage for those who elect to specialize in sports medicine.

Our session on drugs in sports in the preclinical curriculum presented both opportunities and challenges. It successfully introduced an important topic early in medical education, but student feedback and faculty reflection highlighted areas for improvement. The students' comments on the relevance of the material, our approach to instructional blocks, USMLE Step 1 exam preparation, and cultural sensitivities were insightful, especially considering the foundational focus of the preclinical curricula. Traditional lecture hall sessions, whether didactic or team-based activities, generally emphasize learning and application of content-rich, foundational scientific knowledge.

The approach prioritized practical patient engagement skills, including motivational interviewing techniques, instead of content learning. Motivational interviewing skills, while crucial for effective patient care, are less aligned with the usual content delivered to preclinical students in large lecture hall sessions. These skills are typically covered in small group clinical skills sessions held in simulated exam room settings. The session also fell short of conventional approaches to teaching motivational interviewing skills as we did not include role-play, written dialog, or mock-patient counseling techniques (31, 33). The deviation from the norm, while innovative, may have contributed to the challenges identified in student feedback and faculty reflection. The session may be better suited if it was more in line with traditional block content and structured similarly to other case-based team learning sessions that are held in large classroom settings. Additional survey-style session(s) can then be developed to highlight relevant topics (Figure 1) with a clinical skills approach and in small group formats that can be offered in elective or other periods during pre-clinical and clinical years.

## 6 Action plan

Changes are planned for the next iteration of the preclinical session along with more encompassing longer term curriculum planning. The immediate objective will be to realign the learning content with the instructional blocks. Learning objectives will be updated to reflect the stage of student training, desired goals, and planned curriculum redundancy. To address students' focus on the relevance of the content to USMLE Step 1 exams, pertinent doping information can be incorporated into lectures associated with specific drug classes that align with prohibited substances and methods. This would include agents such as anabolic steroids, glucocorticoids, and stimulants, and narcotics (34).

The revised MS1 session will focus on the mechanisms of action including the pharmacology and physiological effects, and side effects of various performance-enhancing drug classes. This approach will build upon and align with other endocrinology lectures, creating a more cohesive learning experience. By intentionally overlapping some content with related lectures, we aim to enhance student engagement and comprehension of these drugs and their actions. This foundational knowledge will enable students to better understand how these substances influence athletic performance. Furthermore, exploring the side effects and performance-enhancing capabilities of these drugs will naturally lead to discussions on the ethical dilemmas these future medical practitioners may face when dealing with athletes or patients using these substances.

Learning objectives will be revised to align with current American Association of Medical Colleges (AAMC) standards, avoiding vague terms like “understanding” and “knowing” (35). Specialized details such as specific drugs on the WADA Prohibited List and the in/out-of-competition status are overly specific for pre-clinical students and will be removed from the session. The curriculum will also de-emphasize topics like the Global Drug Reference Online (GlobalDRO) website (36), therapeutic exemptions, and other aspects of doping control during pre-clinical training, addressing students' valid concerns about the relevance of these topics at their current stage of education.

The revitalized MS2 CBTL session will further build on the introductory pharmacology and doping concepts and provide opportunities for integration of medical knowledge through the cases. A goal will be to highlight materials that are relevant for their upcoming USMLE Step 1 exams. Clinical curriculum presented during the students latter 2 years could then focus on developing the communication skills needed to discuss PED use with patients. This would occur as the students transition to the wards, with additional focus on the ethical dilemma associated with patient drug use. This is especially as athletes are a vulnerable population susceptible to addiction (37, 38). Additional PED cases could be incorporated into the behavior change lectures during 3rd and 4th year clerkships. It would also be appropriate for these clinical students to learn about Global DRO, TUE processes, and other concepts (Figure 1).

Once students are in residency a structured drugs in sports program that is in-line with Graduate Medical Education (GME) content can be designed for students in appropriate specialty pathways such as the Sports Medicine rotation in the Family Medicine Residency Program. Potentially these materials can be drawn from critical elements of the Stanford Continuing Medical Education course, which is designed for practicing physicians who have completed their residency and fellowship training as well as the WADA-ADEL course. The IOC certificate program could also be used for GME with medical residents, however it is far more extensive than the courses offered by Stanford or the ADEL course (20, 29). The more intensive IOC course appears to be best suited for those who will specialize in sports and other practitioners who will work in a team environment and need to be well versed in doping control processes, the biological passport, whereabout testing procedures, and other nuanced issues associated with doping in elite and professional athletes (28, 39–43).

Assessing student learning is essential for refining our curriculum. In this inaugural session, we deliberately chose not to evaluate the outcomes of multiple-choice exams or written group assignments. We were uncertain whether the content was suitable for preclinical students and whether these metrics would provide any meaningful information. Instead, we relied on student survey responses and faculty reflections to gauge whether the session was appropriately structured or if substantial adjustments were needed. Given the extent of revision to the session this was an appropriate avenue to pursue. Moving forward with a revised curriculum more closely tied to the instructional blocks and focused on preparation for USMLE Step 1 exams, we plan to analyze block exam performance and written group reports alongside survey responses to assess student learning and comprehension of the material.

## 7 Conclusions

The inaugural drugs in sports Case-Based Team Learning session for preclinical medical students revealed both potential and challenges in integrating this novel topic into the curriculum. Despite the prevalence of performance-enhancing substances in modern sports, many clinicians lack knowledge in this area. Our session demonstrated that survey-level content is appropriate for preclinical students, but objectives must align with block goals, remain relevant to USMLE Step 1, and be tailored to students' educational stages. Sessions highlighting mechanisms of action of performance-enhancing drugs encompassing the pharmacology and physiology associated with their use, along with related ethical considerations, are suitable for preclinical students. Emphasis should be placed on creating a cohesive learning experience that builds upon existing preclinical lectures, particularly in endocrinology. A well-coordinated preclinical curriculum would provide future physicians with a foundation in drugs in sports, preparing them for various aspects of their careers. This includes training to effectively communicate with patients, address addiction issues, and navigate the complex landscape of drugs in sports. Such foundational knowledge also lays the groundwork for students who may later pursue specialized training in sports medicine. By integrating this topic into the preclinical curriculum, medical schools can better equip future physicians to handle the multifaceted challenges associated with performance-enhancing substances in sports and patient care.

## Data Availability

The original contributions presented in the study are included in the article/supplementary material, further inquiries can be directed to the corresponding author.
